# Impaired Embryonic Development in Mice Overexpressing the RNA-Binding Protein TIAR

**DOI:** 10.1371/journal.pone.0011352

**Published:** 2010-06-28

**Authors:** Yacine Kharraz, Pierre-Adrien Salmand, Anne Camus, Jacques Auriol, Cyril Gueydan, Véronique Kruys, Dominique Morello

**Affiliations:** 1 Laboratoire de Biologie Moléculaire du Gène, Faculté des Sciences, Université Libre de Bruxelles, Gosselies, Belgium; 2 UMR5547, CNRS, CBD, Université de Toulouse, Toulouse, France; 3 Laboratoire de Spécification des Destins Cellulaires chez la Souris, Institut Jacques Monod UMR 7592 CNRS, Université Paris Diderot-Paris 7, Paris, France; 4 Center of Microscopy and Molecular Imaging, Gosselies, Belgium; Centre de Regulació Genòmica, Spain

## Abstract

**Background:**

TIA-1-related (TIAR) protein is a shuttling RNA-binding protein involved in several steps of RNA metabolism. While in the nucleus TIAR participates to alternative splicing events, in the cytoplasm TIAR acts as a translational repressor on specific transcripts such as those containing AU-Rich Elements (AREs). Due to its ability to assemble abortive pre-initiation complexes coalescing into cytoplasmic granules called stress granules, TIAR is also involved in the general translational arrest observed in cells exposed to environmental stress. However, the *in vivo* role of this protein has not been studied so far mainly due to severe embryonic lethality upon *tiar* invalidation.

**Methodology/Principal Findings:**

To examine potential TIAR tissue-specificity in various cellular contexts, either embryonic or adult, we constructed a TIAR transgenic allele (loxPGFPloxPTIAR) allowing the conditional expression of TIAR protein upon Cre recombinase activity. Here, we report the role of TIAR during mouse embryogenesis. We observed that early TIAR overexpression led to low transgene transmission associated with embryonic lethality starting at early post-implantation stages. Interestingly, while pre-implantation steps evolved correctly *in utero*, *in vitro* cultured embryos were very sensitive to culture medium. Control and transgenic embryos developed equally well in the G2 medium, whereas culture in M16 medium led to the phosphorylation of eIF2α that accumulated in cytoplasmic granules precluding transgenic blastocyst hatching. Our results thus reveal a differential TIAR-mediated embryonic response following artificial or natural growth environment.

**Conclusions/Significance:**

This study reports the importance of the tightly balanced expression of the RNA-binding protein TIAR for normal embryonic development, thereby emphasizing the role of post-transcriptional regulations in early embryonic programming.

## Introduction

Post-transcriptional regulations of gene expression play a major role during all phases of organism development and particularly during embryogenesis whose genetic program relies on complex spatio-temporal gene expression patterns. These regulatory processes mostly rely on the recognition of specific *cis*-acting sequences present in messenger RNAs by RNA-proteins and/or non-coding small RNA molecules. TIAR (TIA-1 related) protein belongs to the large family of RNA-binding proteins (RBPs) and is involved in several processes of mRNA metabolism. This protein is composed of three RNA-Recognition Motifs (RRMs) and has been identified by its high degree of similarity to TIA-1 protein. These proteins are both expressed as two isoforms resulting from the alternative splicing of their pre-mRNAs [1]. As numerous RBPs, TIAR and TIA-1 shuttle between the nucleus and the cytoplasm. In the nucleus, they participate in the alternative splicing of several hnRNAs [2]-[4] and are loaded onto mRNA precursors prior to their export to the cytoplasm [5]. In the cytoplasm, TIAR has been shown to regulate the translation of various mRNAs bearing AU-rich elements (AREs) in their 3′ untranslated region (UTR). For example, mRNAs encoding human matrix metallinoproteinase-13 (HMMP13) and β2-adrenergic receptor are translationally repressed by TIAR [6], [7] and *c-myc* mRNA translation is controlled by the competitive binding of TIAR and AUF1, another RBP, to its ARE [8]. Furthermore, microarray analysis of TIAR RNA ligands revealed the capacity of TIAR to bind and regulate the translation of transcripts bearing a C-rich sequence in their 3′ UTR [9]. In addition to the translational regulation of specific mRNAs, TIAR is involved in a broader translational repression mechanism which takes place in cells having to overcome environmental stresses such as UV irradiation, thermic variations or oxidative shock [10]. Thus, though nuclear at steady state in most somatic cells, TIAR exerts both nuclear and cytoplasmic functions. While sharing several structural and functional similarities, specific properties for TIAR and TIA-1 are suggested by the partially diverging phenotypes of mutant mice lacking either of these two proteins. Indeed, while the inactivation of *tiar* and *tia-1* genes both leads to relatively severe lethality, *tiar^−/−^* survivors only suffer from impaired gametogenesis and infertility due to disorders in the development process of primordial germ cells [1], [11]. In the C57Bl6 background, most *tiar^−/−^* embryos die in utero (90%) [1], while none survive in the BalB/c background [11]. Causes of embryonic lethality were not described in these studies and analysis of lethality before E10.5 was not reported, precluding thus any precise knowledge on TIAR requirement in early embyogenesis.

The present study aimed at the characterization of the role of TIAR during mouse embryogenesis using a gain of function approach. We report that TIAR controls late pre-implantation stages and that its overexpression significantly impairs embryonic development beyond implantation, thereby revealing the importance of an adequate TIAR expression level for the physiology of mouse embryo.

## Results

### Generation and characterization of mice carrying a transgene allowing tissue-specific expression of TIAR

**Figure 1:**
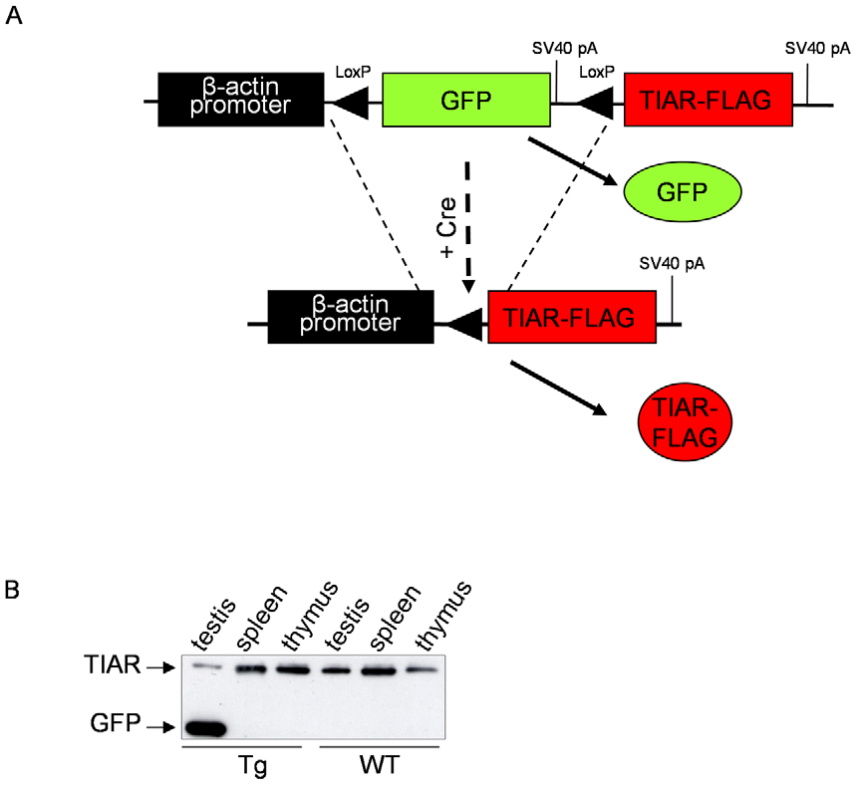
Generation and characterization of mice carrying a transgene allowing tissue-specific expression of TIAR. (A) Schematic representation of the GFP-TIAR transgene and recombination by Cre recombinase. (B) Analysis of transgene expression in mouse tissues by western blot using anti-GFP antibodies. Twenty µg of proteins extracted from the indicated tissues were used for western blot analysis. GFP expression was observed using anti-GFP antibodies and the amount of loaded proteins in the different samples was tested using anti-TIAR antibody (Tg or WT: transgenic or WT tissues respectively).

We first designed a β-actin-TIAR construct (BA-TIAR), in which a sequence encoding a Flag-tagged TIAR short isoform was placed under the control of the β-actin promoter. This construct was injected in fertilized eggs which were reimplanted in pseudo-pregnant females. The injection and reimplantation of 362 eggs led to the birth of 19 individuals, none of which were transgenic. This result is significantly different from our minimal yield of one transgenic out of 5 born individuals, suggesting that TIAR overexpression was embryonic lethal. We thus designed another transgene allowing a conditional expression of TIAR protein based on the insertion of a GFP cassette flanked by LoxP sites between the β-actin promoter and TIAR-Flag coding sequence (Fig. 1A and Fig. S1). This GFP-TIAR construct was used to generate transgenic lines. Three independent founders were obtained out of 238 injected eggs and bred to derive transgenic lines. Two of them (alpha and beta) carried multiple copies (up to 100) of the transgene, the third one (gamma) bearing only 2 to 3 copies (Fig. S2). Transgene expression was analyzed in males of each transgenic line by western blot using anti-GFP antibodies. This analysis revealed that transgene expression was restricted to testis (Fig. 1B for the GFP-TIAR beta line and data not shown). Because this testis-restricted expression pattern was observed in the three GFP-TIAR transgenic lines, we concluded that transgene silencing in somatic tissues would result from the transgene sequence itself and was independent from transgene integration sites into the mouse genome.

### Low transmission correlates with high copy number of the transgene and overexpression of TIAR

**Figure 2:**
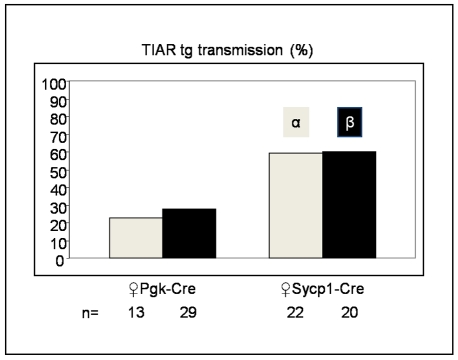
Transmission of the transgene. GFP-TIAR transgene transmission (%) upon mating heterozygous GFP-TIAR males with PGK-Cre or Sycp1-Cre transgenic females. n: number of pups genotyped three weeks after birth using TIAR primers.

To analyze the effects of *tiar* transgene expression, the GFP cassette was deleted *in vivo* by crossing GFP-TIAR males of the alpha and beta transgenic lines with *PGK-cre* transgenic females, which express Cre recombinase during oogenesis [12]. Due to accumulation of Cre recombinase in oocytes, recombination should take place as soon as the transgene included in the male genome is accessible to recombinase, i.e most probably at the pronuclear fusion step. Surprisingly, only 23% and 27.6% of the alpha (n = 13) and beta (n = 29) offspring, respectively, carried the *tiar* transgene, a percentage significantly lower than the 50% expected for the transmission of a heterozygote transgene. This reduced yield of transgenic descendants was not observed upon mating of the same GFP-TIAR males with Scyp1-Cre transgenic females which do not express the Cre recombinase during oogenesis [13] (Fig. 2). Altogether, these results indicate that the reduced transgene transmission is neither due to the sites of transgene integration nor to the transgene sequence itself but most probably results from embryonic lethality due to activation of TIAR transgenic expression upon excision of the GFP cassettes by the Cre recombinase.

**Figure 3:**
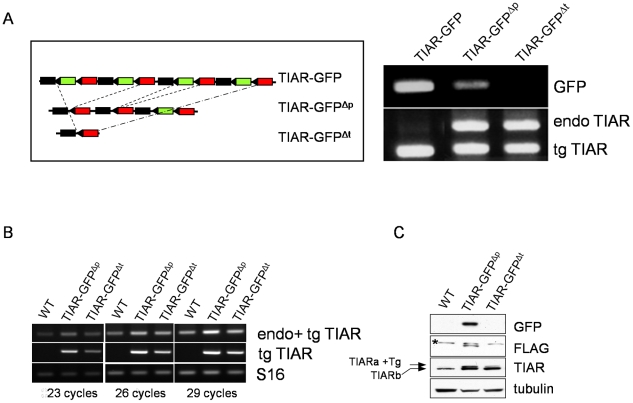
Correlation between transgene copy number and transgene expression. (A) Left: scheme of partial (TIAR-GFP^Δp^) or complete (TIAR-GFP^Δt^) deletion of GFP cassettes upon Cre recombinase activity *in vivo*. Right: PCR amplification from tail DNA of GFP-TIAR, TIAR-GFP^Δp^, TIAR-GFP^Δt^ males with primer sets amplifying GFP or endogenous and transgenic TIAR sequences. (B) *In vivo* expression of TIAR-Flag upon excision of GFP cassettes by PGK-Cre. Semi-quantitative RT-PCR was performed with total RNA from testis of WT, TIAR-GFP^Δp^ and TIAR-GFP^Δt^ mice. Three primer sets were used to detect endogenous and transgenic TIAR (upper lanes), transgenic TIAR (middle lanes) and S16 control mRNAs (lower lanes), respectively. The number of PCR cycles at which a fraction of the PCR reaction was analyzed, is indicated. (C) Western blot analysis of transgenic TIAR expression upon *in vivo* excision of GFP cassettes by PGK-Cre. Twenty µg of protein extracts of testis from the indicated males were loaded on the gel. The indicated antibodies were used to detect transgenic GFP, transgenic TIAR (FLAG) or endogenous TIAR a and b isoforms (TIAR). Asterisk indicates a non-specific band detected by the anti-Flag antibody. Incubation with anti-tubulin antibody indicates that similar amounts of proteins were loaded on the gel.

The viable transgenic mice might be “escapers”, carrying a low copy number of transgene whose weak expression would not preclude embryonic development. This hypothesis suggests that the Cre recombinase excised the GFP cassettes present in the multicopy transgene insertion site at variable efficiency. This will result in the generation of a heterogeneous collection of transgenic embryos containing a wide range of recombined transgene copy number and thus expressing TIAR transgene at variable levels. To evaluate this heterogeneity, we analyzed the presence of GFP cassettes in viable *PGK-Cre* x GFP-TIAR descendants by PCR on tail DNA using GFP-specific primers. The results indeed revealed variability in Cre recombinase activity as GFP cassettes were completely eliminated in some descendants (TIAR-GFP^Δt^) while being only partially eliminated in others (GFP-TIAR^Δp^) (Fig. 3A). While the amplification of TIAR transgene totally competed the amplification of TIAR endogenous gene due to the high copy number of transgene in the GFP-TIAR parental strain, this competition was clearly attenuated in DNA samples from viable (*PGK-Cre* x GFP-TIAR) pups, correlating with a reduction of TIAR transgene copies upon Cre-mediated recombination.

The correlation between the transgene copy number and transgene expression level was assessed by analysing the testis of viable descendants. Semi-quantitative RT-PCR assays revealed that TIAR transgene expression was markedly higher in RNA extracted from testis of partially (TIAR-GFP^Δp^) recombined individuals than in testis of fully (TIAR-GFP^Δt^) recombined ones (Fig. 3B, tg TIAR). Higher expression was confirmed by the use of another couple of primers which amplifies both endogenous and transgenic TIAR mRNAs (Fig. 2B, endo + tg TIAR). Correlatively, higher levels of transgenic TIAR protein were detected in TIAR-GFP^Δp^ testis than in TIAR-GFP^Δt^ testis (Fig. 3C). These data indicate that partial excision of GFP cassettes leads to higher expression levels of transgenic TIAR protein than their complete deletion. When applied to embryonic development, this reasoning implicates that embryos containing partially excised transgenes would express higher levels of transgenic TIAR protein than those containing only one excised copy of the transgene (TIAR-GFP^Δt^, Fig. 3A), the latter being able to fully develop while the former would die.

### Impaired development of TIAR overexpressing embryos at post-implantation stages

**Figure 4:**
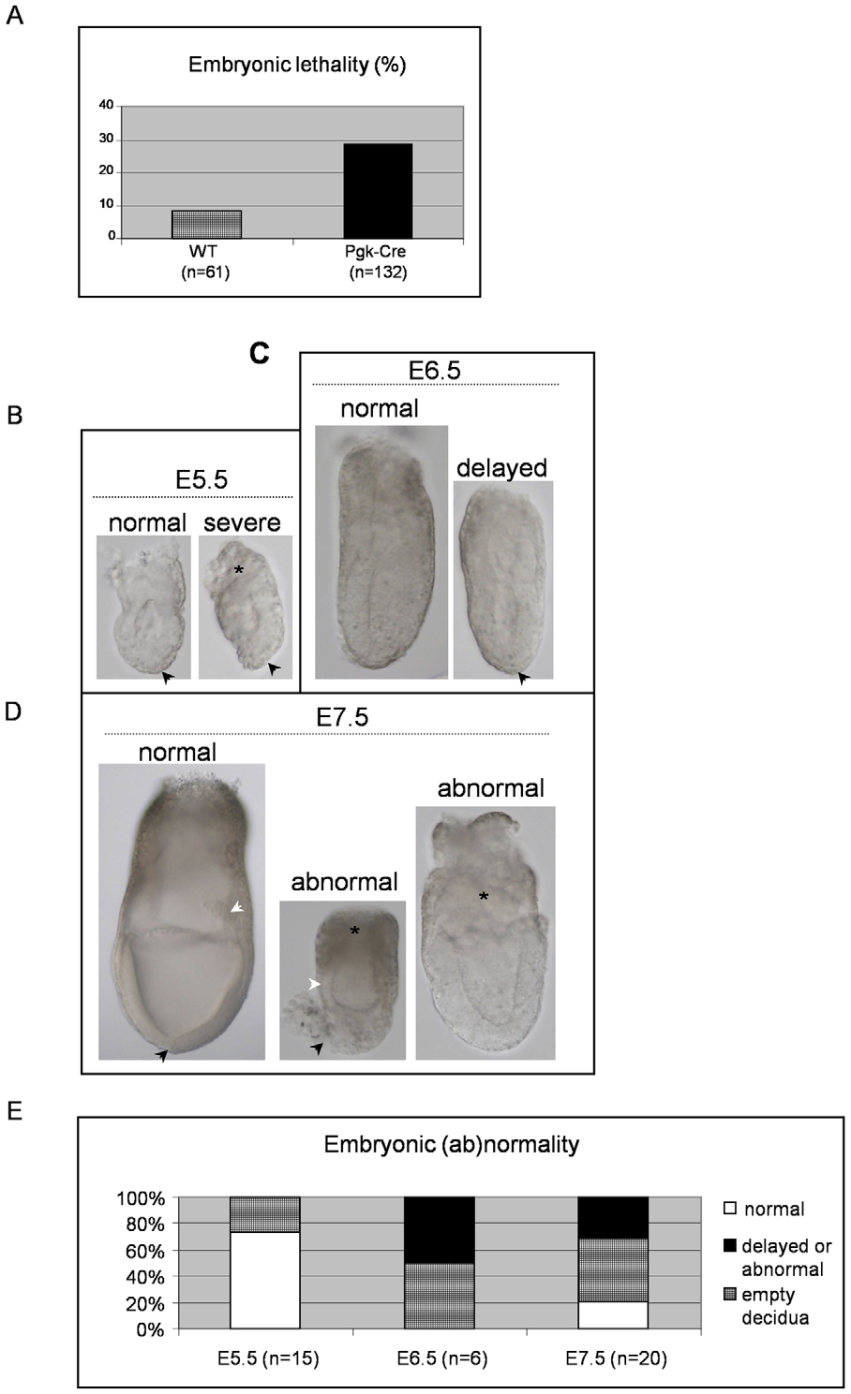
Impaired development of TIAR overexpressing embryos at post-implantation stages. (A) Embryonic lethality within descendants from crossing GFP-TIAR males with WT or *PGK-Cre* expressing females. The values correspond to the % of empty decidua or abnormal embryos found in pregnant *PGK-Cre* or WT females mated with GFP-TIAR transgenic males between 9.5 and 17.5 d*pc*. n: number of counted decidua. (B-D) Representative pictures of post-implantation development of GFP-TIAR x *PGK-Cre* embryos collected between 5.5 d*pc* and 7.5 d*pc* from pregnant *PGK-Cre* females mated with heterozygous GFP-TIAR males. Magnification 20× for all embryos but 10× for the normal E7.5 embryo shown in D on the left. (B) Left: pre-streak stage embryo. Note that the distal VE has not started to shift proximally (arrowhead). The anterior movement of distal VE cells converts Proximal-Distal polarity to Anterior-Posterior polarity in the pregastrula embryo; Right: pre-streak stage embryo with abnormal thickening of the VE (arrowhead) and reduced ExE (asterisk). (C) Left: Gastrulation has started in this early-streak stage embryo with the formation of the primitive streak posteriorly; Right: pre-streak stage embryo with distal VE (arrowhead). (D) Left: Gastrulation is completed and the three germ layers are established in this early-neural-plate stage embryo. The primitive streak has extended to reach the distal tip of the embryo where a node has formed (black arrowhead). The amnion is closed and an allantois bud is visible (white arrowhead). Middle: Abnormally small embryo with a reduced ExE (asterisk), thin-looking epiblast layer (white arrowhead) and expanded pro-amniotic cavity. Note distally the abnormal accumulation of the VE (black arrowhead); Right: Abnormally small embryo with a reduced ExE (asterisk) and no visible primitive streak or node. (E) Post-implantation lethality. Embryos from pregnant *PGK-Cre* females mated with homozygous GFP-TIAR males were collected between 5.5 and 7.5 d*pc* and the number of empty decidua, delayed/abnormal or normal embryos was counted. n: number of analyzed decidua.

To test this hypothesis, we compared the development of embryos resulting from the mating of heterozygous GFP-TIAR transgenic males with WT or *PGK-cre* females. Embryos were collected between 9.5 and 17.5 day *post coitum* (d*pc*) and the number of normal versus abortive embryos (or empty decidua) was counted. As shown in figure 4A, embryonic lethality was significantly higher (29%) when GFP-TIAR transgenic males (from both α and β strains) were mated with *PGK-cre* females than with WT females (8 %). This result *a posteriori* explains why we obtained a reduced number of living transgenic pups from such crosses or none in our first attempt with the BA-TIAR construct and reinforces the conclusion that TIAR overexpression is incompatible with normal embryonic development.

We further investigated the embryonic stage at which *PGK-cre* x GFP-TIAR embryos degenerated *in utero* by recovering embryos at earlier developmental stages. After implantation, the rapid growth of the epiblast (derived from the inner cell mass), the extraembryonic ectoderm (derived from the trophectoderm) and the overlaying visceral endoderm (derived from the primitive endoderm) leads to the formation and elongation of an egg-cylinder-shaped pre-streak embryo [14], [15]. The visceral endoderm (VE) and extra-embryonic ectoderm (ExE) have been shown to play an important role in the regulation of the growth and patterning of the epiblast [16], [17]. Proliferation and morphogenetic movements within the epiblast layer cooperate for the initiation of the primitive streak and the formation of the three embyonic germ layers [18].

On the basis of morphological criteria [19]–[21], we analysed the development status of 5.5 to 7.5 d*pc* embryos resulting from the mating of GFP-TIAR heterozygous males (from both α and β strains) with *PGK-Cre* expressing females. Theoretically, all oocytes of such females (either heterozygous or homozygous) express the Cre recombinase which is able to act on the paternal TIAR floxed allele transmitted in 1 out of 2 embryos. We could thus compare within the same litter the embryonic development of WT and TIAR transgenic embryos. While most E5.5 embryos of the same litter appeared normal (Fig. 4B), we observed an increase in the number of developmentally delayed or abnormal embryos (as illustrated in Fig. 4C–D) with age. To know whether retarded or degenerating embryos were transgenic, we either cultivated the ectoplacental cone (EPC) of E5.5–6.5 embryos or directly extracted DNA from yolk sac (E7.5) to perform a semi-quantitative or quantitative PCR analysis using TIAR primers. We observed that yolk sac or EPC from apparently normal embryos were either not transgenic or contained 1–2 copies of transgene while those from degenerating or retarded embryos had 32–60 copies of transgene (data not shown). To get a more quantifiable analysis of embryonic lethality between 5.5 and 7.5 d*pc*, we repeated the above mentioned experiment starting with homozygous instead of heterozygous GFP-TIAR α and β males. We observed that the percentage of delayed and abnormal embryos increased between 5.5 and 7.5 d*pc*, the number of apparently normal embryos going from 80% at 5.5 d*pc* to 23% at 7.5 d*pc* (Fig. 4E). Altogether, these results indicate that the development of *PGK-Cre* x GFP-TIAR embryos is severely compromised rapidly after implantation as embryonic viability decreases as early as E5.5.

### Increased sensitivity of PGK-Cre x GFP-TIAR blastocysts to *in vitro* culture

**Figure 5:**
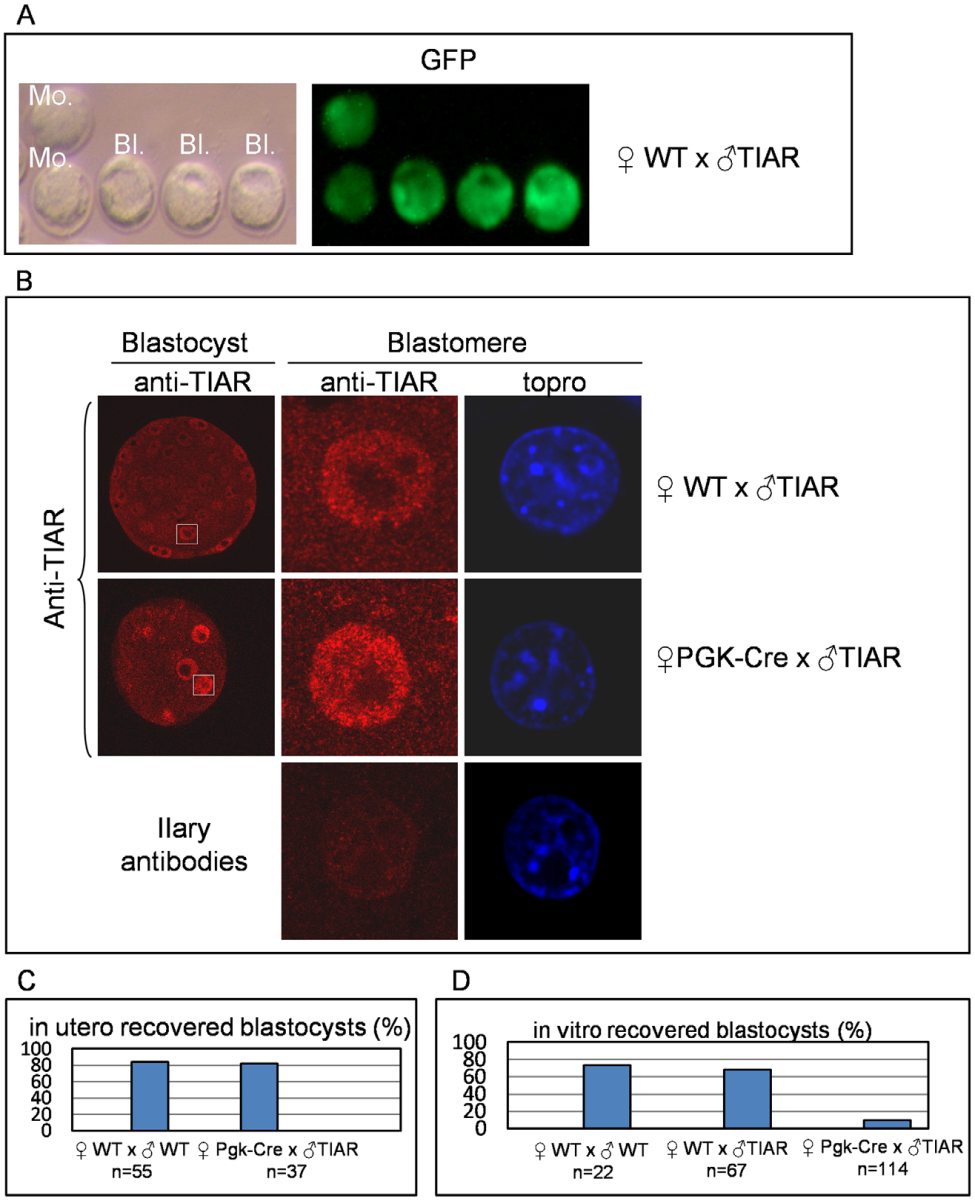
*In utero* vs *in vitro* development of TIAR overexpressing embryos. (A) Pre-implantation embryos from pregnant WT females mated with homozygous GFP-TIAR males were collected at various pre-implantation stages and observed for GFP expression. GFP expression starts at morula (mo) stage and persists at the early blastocyst stage (bl) where the blastocoele cavity starts to be clearly visible when observed upon bright field. (B) E3.5 embryos were collected from uterus of WT or *PGK-Cre* females mated with homozygous GFP-TIAR (TIAR) males, stained for TIAR and observed under confocal microscope using TOPRO to visualize the nucleus of each blastomere. The first column shows representative pictures of blastocysts while the second column shows blastomere magnification. IIary antibodies correspond to embryos for which the first step of labelling (anti-TIAR antibody) has been omitted to assess background fluoresence. (C) E3.5 embryos were collected from uterus of WT or *PGK-Cre* females mated with WT or homozygous GFP-TIAR (TIAR) males and the % of “healthy” blastocysts with no obvious fragmentation was counted. (D) Embryos from WT or *PGK-Cre* females mated with WT or homozygous GFP-TIA (TIAR) males were collected at E0.5 or E2.5, cultured in M16 medium until WT embryos hatched (for 2-4 days) and the % of “healthy” blastocysts with no obvious fragmentation was counted. n: number of counted embryos.

Empty deciduas (33%) are observed as early as 5.5 d*pc* suggesting that TIAR could have been accumulated during the pre-implantation stages resulting in embryonic lethality shortly after implantation. To verify this hypothesis, we first analysed the stage at which TIAR accumulation could start taking first GFP as a reporter of β-actin promoter activity in embryos recovered from WT x GFP-TIAR crosses. GFP expression was detectable from the morula stage and beyond (Fig. 5A). Thus, after Cre recombination, the transgenic TIAR protein is not expected to be expressed before morula stage. Unfortunately, we could not analyse specifically TIAR transgenic expression due to high background signal when using anti-FLAG antibody. We therefore determined whether TIAR was overexpressed in transgenic embryos by immunofluorescence analysis of *in utero* collected blastocysts from WT or *PGK-Cre* females mated with homozygous GFP-TIAR (α and β strains). We observed by confocal microscopy that *PGK-Cre*-derived blastocysts were indeed more stained than the WT ones with a strong staining of the nucleus of each blastomere (Fig. 5B).

**Figure 6:**
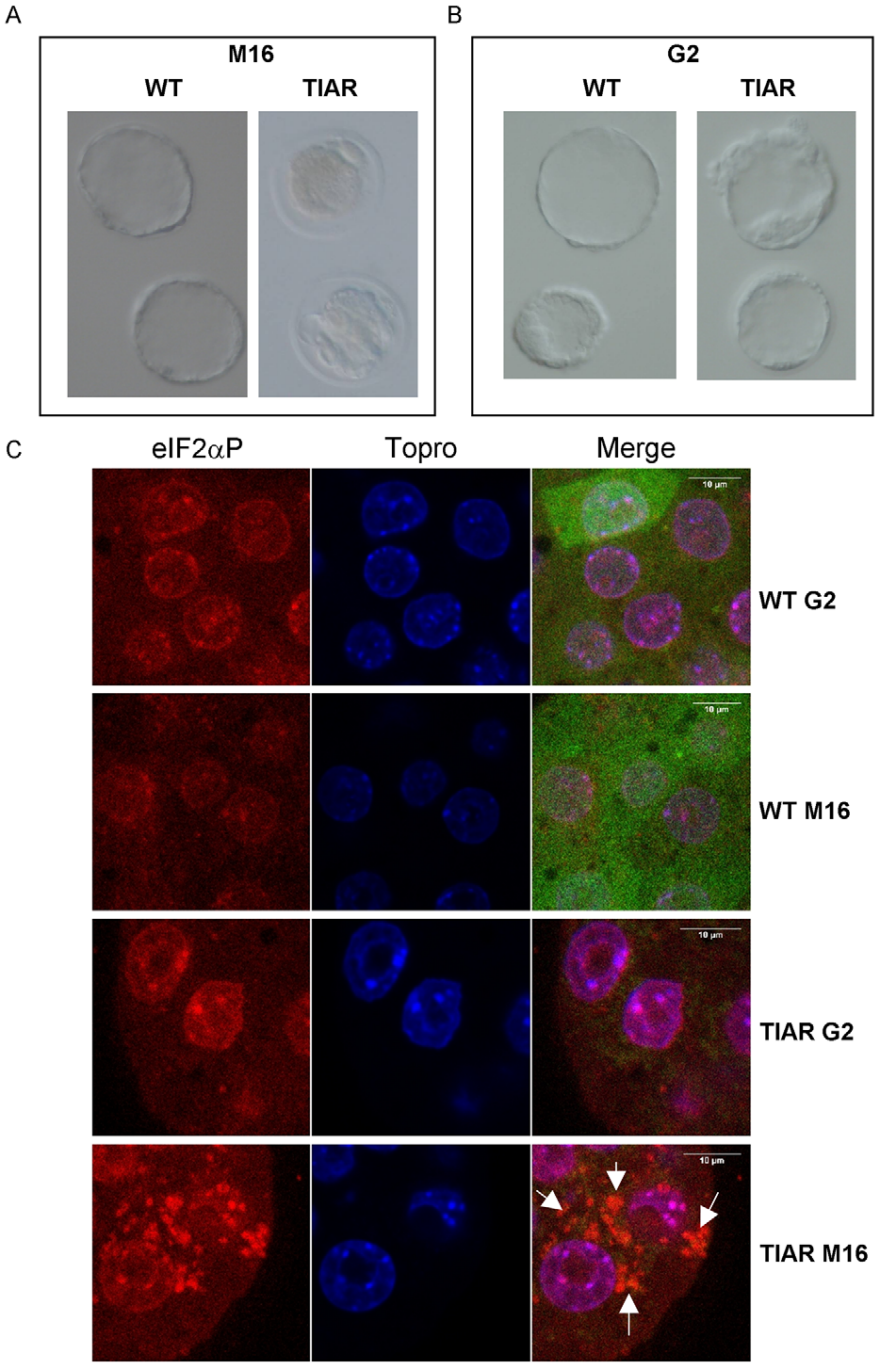
Increased sensitivity of *PGK-Cre* x GFP-TIAR blastocysts to *in vitro* culture correlated with accumulation of phospho-eIF2α in cytoplasmic granules. (A,B) Representative pictures of embryos from WT or *PGK-Cre* x GFP-TIAR (TIAR) crosses grown *in vitro* in M16 (A) or G2 (B) medium until they reach hatched blastocyst stage. A. in M16 fragmentation and unevensized blastomeres are observed in TIAR compared to healthy looking hatched WT blastocysts. In G2 medium the TIAR embryos are not distinguishable from WT ones (B). (C) WT or *PGK-Cre* x GFP-TIAR (TIAR) embryos cultured from 8-cell stage to blastocyst stage in G2 or M16 medium were fixed and stained with anti-phospho-eIF2α antibody and TOPRO to label nuclei and observed by confocal microscopy. WT embryos express GFP while GFP has been deleted in Cre-expressing embryos (TIAR), as shown in merge pictures. While artefactual phospho-eIF2α staining due to TOPRO labelling is observed in the nuclei of all types of embryos, phospho-eIF2α accumulates only in the cytoplasm of TIAR embryos grown in M16 medium. Shown are representative images obtained from two independent experiments where 5–20 embryos of each categories were analyzed. The scale bars represent 10 µm.

Such TIAR overexpression has no apparent detrimental consequences on *in utero* pre-implantation development since healthy blastocysts were recovered *in utero* with the same yield from WT or *PGK-Cre* females mated with homozygous GFP-TIAR α and β males (Fig. 5C). Then, we analysed the ability of TIAR overexpressing embryos to develop *in vitro*. Fertilized one-cell stages or morula recovered from WT or *PGK-Cre* females were cultured in M16 medium. We observed a low rate of blastocyst formation for the *PGK-Cre*-derived embryos compared to WT, as assessed by the number of blastocysts with even-sized blastomeres with no obvious fragmentation (Fig. 5D, 6A). Surprisingly, when cultured in the highly specialized G2 medium, most morula from *PGK-Cre* females developed into fully hatched blastocysts and no difference was observed in their ability to reach this stage when compared to WT derived-embryos (Fig. 6B). We then investigated whether the lethality of TIAR overexpressing embryos in M16 medium was due to cellular stress by measuring the phosphorylation status of the translation initiation factor eiF2α [22], [23]. The sensitivity of PGK-cre-derived pre-implantation embryos to M16 medium was correlated with a strong accumulation of the phosphorylated form of eiF2α in their cytoplasm. Cytoplasmic concentration of phospho-eiF2α was observed neither in transgenic embryos grown in G2 medium nor in WT embryos either grown in M16 or G2 medium (Fig. 6C). Altogether, these results indicate that TIAR-overexpressing embryos are much more sensitive to cell culture conditions than WT ones. Depending on favourable (*in utero* or G2 medium) or stressed conditions (M16), TIAR accumulation either does not interfere or prevents further development.

## Discussion

TIAR protein has been described as a major factor intervening in several post-transcriptional gene regulatory processes both in physiological and stress conditions [24], [25]. In the present study, we wished to investigate its role during early embryogenesis. Owing that *tiar* inactivation is embryonic lethal [1], [11], a conditional knock out allele would have been an appropriate tool to study this question. However, since this strategy is rather time consuming and would have necessitated a battery of Cre-expressing mice, we have decided to use the complementary approach of gain of function and used a conditional GFP floxed-*tiar* transgene to overexpress a Flag-TIAR protein. Although such a strategy has its own limitation that resides mainly in the inability to control the extent of overexpression, it nevertheless allowed us to demonstrate that adequate TIAR expression level is required for proper early embryonic development. Observation of both weak transgene transmission, most probably resulting from embryonic lethality, and transgenic survivors led us to postulate that Cre efficiency was variable from one embryo to another. This hypothesis was reinforced by experiments aimed at measuring both transgene copy number and transgene expression that indicated that when the Cre recombinase was fully or almost fully efficient leading to one or a few copies of transgenes, transgenic embryos survived and gave rise to survivors expressing transgenic TIAR protein at a weak level (no more than twice the endogenous level in testis). Reversely, embryonic lethality correlated with a higher transgene copy number, strongly suggesting that when the Cre is partly efficient, the GFP cassette is excised from multiple transgene copies, resulting in a *tiar* transgene expression above the threshold level compatible with embryonic survival.

The analysis of GFP expression in embryos derived from GFP-TIAR males crossed with wild-type females revealed that transgene expression driven by the β-actin promoter is activated from the morula stage. Consequently, after Cre recombination, transgenic TIAR might accumulate from this stage and beyond. Microarray analysis of mouse pre-implantation development indicates that *tiar* gene is expressed during pre-implantation stages [26] and its expression increases from the 8-cell stage mouse embryo [27], coincidently with the second wave of transcription, named mid-preimplantation gene activation (MGA), that precedes the dynamic morphological and functional changes from the morula to blastocyst stage [26], [28]. At the protein level, our confocal analysis revealed that TIAR protein is expressed during pre-implantation stages and that, due to transgene expression, TIAR is more expressed in (*PGK-Cre* x GFP-TIAR)-derived blastocysts than in WT or (WT x GFP-TIAR) age-matched control ones. Such TIAR accumulation, mainly in the nucleus, did not apparently perturb *in utero* pre-implantation development since healthy blastocysts were recovered with the same yield from WT or *PGK-Cre* females. However, TIAR blastocyst formation was compromised in the sub-optimal M16 culture medium where no blastocysts were recovered. By contrast, in the serum-free, chemically defined G2 medium designed to enhance development to the blastocyst stage of human cleavage stage embryos [29], [30], TIAR embryos recovered from either WT or *PGK-Cre* females developed to the expanded blastocyst stage and all blastocysts were hatching from the zona pellucida. This observation led us to suggest that the overexpressing TIAR genome may be more susceptible to environmental cues related to growth conditions than the wild type genome. This hypothesis was verified by studying the phosphorylation status of the translation initiation factor eIF2α. We found that phospho-eIF2α expression was strongly and specifically increased in *PGK-Cre*-derived blastocysts grown in M16 medium. Phosphorylation of eIF2α is a well-known consequence of environmental stresses and results in the general blockade of protein synthesis [22], [23]. TIAR misexpression might thus sensitize embryos to sub-optimal growth conditions, promoting eiF2α phosphorylation and subsequent inhibition of protein synthesis through mechanisms that remain to be fully defined (see below). Susceptibility to environment has been previously reported for the H19 gene whose expression in mouse zygotes could be experimentally manipulated by *in vitro* culture conditions [31] and for reprogrammability of somatic nuclei in mouse clones [32].

Accumulation of TIAR protein does not seem to preclude blastocyst implantation in the endometrium since no difference in the number of decidua was detected between the different crosses. However, empty decidua were observed at a higher frequency in *PGK-Cre* females than in WT females at all post-implantation stages analysed. In addition, our morphological analysis from 5.5 to 7.5 d*pc* reveals that TIAR embryos with a high transgene copy number are developmentally delayed or severely compromised. They failed to establish the anterior-posterior axis, or to initiate gastrulation and to form the three embryonic germ layers. Altogether, these observations, in particular the increased sensitivity of pre-implantation embryos to growth culture conditions and the decreased embryonic viability observed from 5.5 d*pc* strongly suggest that TIAR accumulation from blastocysts is not compatible with post implantation development.

How TIAR overexpression may sense the extra-cellular environment and what are the TIAR molecular targets are important and yet partly unsolved questions. Theoretically, TIAR overexpression could affect development either through impaired expression of a few genes or induce wide gene expression disorders such as the one encountered in *in vitro* cultured somatic cells submitted to environmental stresses [10]. Following UV irradiation, thermic variations or oxidative shock, TIAR accumulates in so-called stress granules (SGs) that contain 43S pre-initiation complexes corresponding to aggregates of capped and polyadenylated mRNAs associated with small 40S ribosomal subunits. SG formation is concomitant with inhibition of translation of numerous mRNAs [33], [34]. To know whether SGs form upon TIAR overexpression, we analysed the cytoplasmic distribution of two routinely used markers of SGs, eIF3b, a component of the pre-initiation complex [35], and eIF2α whose phosphorylation initiates the assembly of TIAR-containing SGs [10], [23]. We did not observe any obvious difference between *in utero* collected WT and (*PGK-Cre*xTIAR) blastocysts, in particular eIF3 did not accumulate in discrete cytoplasmic foci in *in utero* developing transgenic blastocysts (data not shown). In striking contrast, when transgenic embryos were grown in M16 medium, phosphorylation of eIF2α increased dramatically as mentioned above and phospho-eIF2α accumulated in cytoplasmic granules. Altogether, our data strongly suggest that depending on growth context, TIAR overexpression either leads to a general inhibition of protein synthesis via its ability to induce SGs and does not permit blastocyst formation *in vitro*, or specifically targets a subset of mRNAs whose altered expression compromises further peri- or post-implantation development.

**Figure 7:**
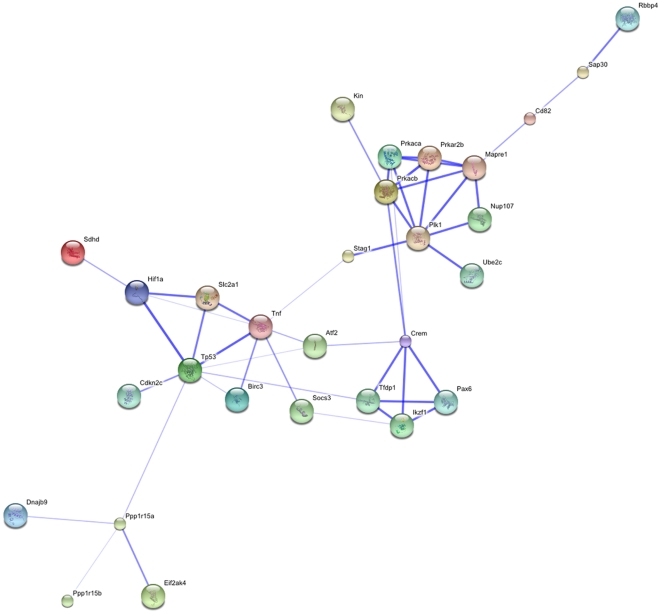
Potential associations between proteins encoded by TIAR-associated mRNAs and expressed during pre-implantation stages. Interconnection revealed using String database (http://string.embl.de/) between 29 proteins encoded by mRNAs specifically bound by TIAR and which are expressed in pre-implantation development.

One classical approach to identify those targets would be to immunoprecipitate mRNAs associated with TIAR. However, the scarcity of preimplantation materials hampers such an experimental strategy. To get around this difficulty, we switched to the macrophage-like RAW264.7 cell line, currently used to address TIAR function in hematopoiesis. RAW264.7 cells stably expressing the very same Flag-tagged TIAR transgene or a RNA-binding defective TIAR mutant were generated and were used for RIP-ChiP analysis. This approach led to the identification of 779 mRNAs specifically bound by TIAR but not by the RNA-binding defective mutant among which was found known TIAR ligands such TNF-α mRNA [1], [36], [37] (Kharraz et al., in preparation). We then crossed this list of TIAR-associated mRNAs with the list of mRNAs expressed during pre-implantation stages (http://lgsun.grc.nia.nih.gov/microarray/data.html) [26] and ended up with 141 mRNAs. Those mRNAs were submitted to an analysis enabling to establish the potential associations, either direct (physical) and/or indirect (functional), between the corresponding proteins (http://string.embl.de/). Twenty nine proteins including TNF-α, were found interconnected in a network centred on Trp53, Pax 6 and cAMP-dependent protein kinase (PKRA) (Fig. 7). The list of the corresponding 29 mRNAs (Table 1) is significantly enriched in ARE-containing mRNAs since 37% of them -instead of the generally admitted 5-8% abundance [38]- contain the WWWU(AUUUA)UWWW sequence used to construct the ARE database (http://brp.kfshrc.edu.sa/ARED/). Remarkably, the list contains *Myd116/Ppp1r15a* and *Ppp1r15b* genes that both encode proteins recruiting phosphatase catalytic subunits of the PPP1 class to phospho-eiF2α [39], [40]. One can thus speculate that TIAR overexpression could attenuate Ppp1r15a/b levels, thereby favouring eiF2α phosphorylation in stressful conditions. It is worth noting that TIAR overexpression in M16 grown embryos phenocopies the double knock-out of *Ppp1r15a* and *b*, as both induce blastocyst E3.5 lethality *in vitro* [41].

**Table 1:** List of functionally or physically interconnected proteins encoded by mRNAs specifically immunoprecipitated by TIAR in RAW264.7 cells and expressed in pre-implantation development.

Groups	Accession number	Symbol	Gene name	ARE cluster
Trp53/TNF-α	NM_011640	Trp53	transformation related protein 53	No ARE
	NM_013693	Tnf	tumor necrosis factor	Cluster 3
	NM_007707	Socs3	suppressor of cytokine signaling 3	Cluster 5
	NM_010431	Hif1a	hypoxia inducible factor 1, alpha subunit	Cluster 3
	NM_011400	Slc2a1	solute carrier family 2 (facilitated glucose transporter), member 1	Cluster 5
	NM_013719	Eif2ak4	eukaryotic translation initiation factor 2 alpha kinase 4	No ARE
	NM_025848	Sdhd	succinate dehydrogenase complex, subunit D	No ARE
	NM_007671	Cdkn2c	cyclin-dependent kinase inhibitor 2C (p18, inhibits CDK4)	No ARE
	NM_008654	Ppp1r15a	protein phosphatase 1, regulatory (inhibitor) subunit 15a	Cluster 1
	NM_133819	Ppp1r15b	protein phosphatase 1, regulatory (inhibitor) subunit 15b	No Are
	NM_007464	Birc3	baculoviral IAP repeat-containing 3	No ARE
	NM_013760	Dnajb9	DnaJ (Hsp40) homolog, subfamily B, member 9	No ARE
Pax6	NM_009715	Atf2	activating transcription factor 2	Cluster 5
	NM_013498	Crem	cAMP responsive element modulator	No ARE
	NM_013627	Pax6	paired box gene 6	Cluster 5
	NM_009361	Tfdp1	transcription factor Dp 1	No ARE
	NM_009578	Ikzf1	zinc finger protein, subfamily 1A, 1 (Ikaros)	No ARE
Prka	NM_021788	Sap30	sin3 associated polypeptide	No ARE
	NM_009030	Rbbp4	retinoblastoma binding protein 4	Cluster 5
	NM_026785	Ube2c	ubiquitin-conjugating enzyme E2C	No ARE
	NM_011121	Plk1	polo-like kinase 1 (Drosophila)	No ARE
	NM_011100	Prkacb	protein kinase, cAMP dependent, catalytic, beta	No ARE
	NM_007656	Kai1	kangai 1 (suppression of tumorigenicity 6, prostate)	No ARE
	NM_025280	Kin	antigenic determinant of rec-A protein	No ARE
	NM_011158	Prkar2b	protein kinase, cAMP dependent regulatory, type II beta	Cluster 5
	NM_134010	Nup107	nucleoporin 107	No ARE
	NM_009282	Stag1	stromal antigen 1	Cluster 5
	NM_008854	Prkaca	protein kinase, cAMP dependent, catalytic, alpha	No ARE
	NM_007896	Mapre1	microtubule-associated protein, RP/EB family, member 1	No ARE

The list of TIAR RNA ligands was generated from RIP-Chip data performed in triplicate by immunoprecipitating stably expressed TIAR-Flag or RRM2-lacking TIAR-Flag mutant with anti-Flag-coupled sepharose beads. RNAs binding full-length but not truncated TIAR protein were included in the list, several of which were confirmed by RIP-qRT-PCR.

Besides those two potential TIAR targets, we noted a high proportion of genes involved in signalling pathways (e.g. TNF-α and HIF-α, table 1), leading us to suggest the attractive hypothesis that through the coordinated control of those pathways, TIAR could allow modifications of RBPs, such as TIAR itself or other RBPs, like HuR, KSRP or ZFP36, leading either to reduced binding to their target mRNAs and subsequent increased mRNA stability or, reversely, decreased stability and/or translation [42], [43].

In summary, our study strengthens the hypothesis that the control of gene expression during preimplantation development is not solely contributed by changes in gene transcription but relies on the regulation of transcript stability and translation [44]. Up to 380 RBPs have been identified in the mouse genome [45] but very few studies have been aimed at characterizing their role during mammalian early development [44]. Our analysis highlights the major role played by TIAR in early embryonic development and indicates that TIAR represents a pivotal actor to control undesired changes in preimplantation embryo and fetal programming.

## Materials and Methods

### Ethics statement

Animal care and experimental procedures were carried out in accordance with the Belgian law of August 14th, 1986 as well as the royal decree of November 14th, 1993 on the protection of laboratory animals for IBMM (ULB)and were approved by the Service public federal de sante publique, securite de la chaine alimentaire et environnement(Direction générale Animaux, Végétaux et Alimentation) (laboratory licence # LA 1500474). Mice were maintained in the mouse colony, checked daily on a regular basis and euthanasized for embryo recovery, according to the french government recommendations (www.legifrance.gouv.fr/) in the CBD (Universite Paul Sabatier and CNRS) and were approved by the Prefecture de la Haute Garonne - France(# agreement genetically modified animal: 4992).

### Genotyping and quantitative analysis of transgene copy number

PCR analyses were carried out using genomic DNA, which was extracted from the tails of wild-type or transgenic mice. Transgene detection was carried out by PCR (30 cycles, hybridization temperature: 52°C) using primers amplifying a 500 bp-TIAR fragment including an intronic sequence in the endogenous gene and a 240 bp-fragment from the transgene (forward primer: 5′GAAGGACATGTGGTGAAATG3′; reverse primer: 5′TCCAAATCCTTGTTGGTTCC3′). Cre recombinase transgene (PGK-Cre or Scy-Cre) was detected by PCR (30 cycles; hybridization temperature: 59°C) using Cre recombinase-specific primers (forward primer: 5′TGATGGACATGTTCAGGGATC3′; reverse primer: 5′CAGCCACCAGCTTGCATGA3′). The presence of residual GFP cassettes in GFP-TIAR x PGK-Cre mice was analyzed by PCR (30 cycles, hybridization temperature: 60°C) leading to amplification of a 200 bp-fragment from the GFP sequence (forward primer: 5′GACGTAAACGGCCACAAGTT3′; reverse primer: 5′AAGTCGTGCTGCTTCATGTG3′). The band intensity revealed partial or total excision of GFP cassettes by the Cre recombinase. Comparative analysis of transgene copy number in embryos was performed on DNA extracted from yolk sac or EPC (ectoplacental cone) by quantitative PCR using MyiQ Bio-Rad apparatus and the following primers: forward: 5′GATGGGTGGATTTGGTGCTC3′ and reverse: 5′TCACTGCATTCTAGTTGTGG3′.

### Semi-quantitative RT-PCR analysis of transgene expression

Total RNA was extracted from mouse tissues using Trizol reagent (Invitrogen) according to the manufacturer's instructions. Endogenous and transgenic TIAR mRNA accumulation was analyzed by semi-quantitative RT-PCR. Single-stranded cDNA was synthesized from 2 µg of total RNA using SuperScript reverse transcriptase (Invitrogen) in the presence of oligodT. One µl of the 20 µl RT reaction was used for semi-quantitative PCR with the same primers used for genotyping analysis and which allow the amplification of both the endogenous and the transgenic mRNAs. Transgene-specific TIAR mRNA was detected by using TIAR forward primer and a reverse primer hybridizing in the SV40 3′UTR (5′TTTTCACTGCATTCTAGTTGTGGTT3′). PCR amplification of the cDNA encoding S16 ribosomal protein was performed as internal control. 13 µl of the PCR reactions (initial volume of 100 µl) were taken after 23, 26, and 29 cycles. PCR products were analyzed by electrophoresis on 1.5% agarose gels.

### Protein extraction and western blot analysis

Protein extraction and western blot analyses were carried out as described previously [46]. The following antibodies were used: mouse monoclonal anti-TIAR 6E3 (dilution: 1/1000; kind gift of N. Kedersha, Harvard), mouse monoclonal anti-Flag M2 (dilution: 1/1000; Sigma), anti-tubulin (dilution: 1/1250; Sigma).

### Embryo experiments

WT (CBA x C57Bl/6) and *PGK-Cre* superovulated females were bred with heterozygous or homozygous GFP-TIAR males. The day of the vaginal plug was considered to be as day 0.5 of pregnancy. One-cell stage fertilized embryos were recovered from the swollen ampulla 17 h–20 h after hCG injection. The embryos were briefly treated with hyaluronidase (1 mg/ml) and further cultured in microdrops of Whitten medium (M16). Morula (E2.5) or blastocysts (E3.5) were flushed from oviducts or uterus, respectively, and grown in M16 or G2™ (Vitrolife/JCD) covered with paraffin oil and incubated at 37°C in a humidified atmosphere of 5% CO2 in air until the time of observation. E5.5 to E17.5 post-implantation embryos were recovered and staged according to Down and Davies [19] and Kaufman [20]. By convention, in figures the post-implantation embryos are shown anterior to the left.

For the fluorescent microscopy analysis, E3.5 embryos were recovered from uterus, briefly grown in M16 medium before being fixed in 4% (V/V) paraformaldehyde in PBS for 10 minutes, washed in M2 medium, permeabilized with Triton X100, 0,1% in PBS for 10 min at RT and incubated 5 min. in PBT (PBS, 3% BSA, 0,1%Tween 20). TIAR, eIF3η or phospho-eIF2α specific labelling was performed using goat anti-TIAR anti-eIF3η polyclonal antibody (C18, or N-20, respectively, Santa Cruz) or anti-Phospho eIF2α (Ser51) (Cell Signaling Technology) at a dilution of 1/50 for 1 h RT. After 3 washes in PBT, donkey anti goat-Alexa 488 or anti rabbit-Alexa 555secondary antibody was added for 45 min at RT. Embryos were washed 3 times in PBT and their nuclei stained by 30 min. incubation in TOPRO (Molecular Probes) (1/200 in M2) at RT in dark. Embryos were rinsed twice in PBT, mounted in Vectashield (Vector Labs) and observed under a Leica SP2 Confocal microscope equipped with helium neon lasers and appropriate filter combination.

## Supporting Information

Figure S1 Characterization of TIAR transgene. (A) Transgene recombination in Cre recombinase-expressing bacteria. Plasmid DNA was transformed and amplified in wild-type or Cre recombinase-expressing bacteria (294-Cre) (gift of S. Schurmans, ULB). Plasmid DNA was then isolated and analyzed by agarose gel electrophoresis after cleavage by Hind III restriction enzyme. The low molecular weight fragment generated by HindIII cleavage is 1385 bp shorter in Cre recombinase-expressing bacteria (lane 2) than with plasmid DNA isolated from wild-type bacteria (lane 1). The size (bp) of the fragments composing the ladder is indicated. (B) Plasmid DNAs amplified in wild-type or Cre recombinase-expressing bacteria were transiently transfected into COS cells using Fugene-6 (Roche) according to the manufacturer's instructions. Cells were harvested 48 h after transfection and lysed for western blot analysis of TIAR-Flag expression with anti-Flag antibody (upper panel). Twenty µg of cell extract was loaded on the gel. The membrane was subsequently incubated with anti-actin antibody to control gel loading (lower panel).
(3.34 MB TIF)

Figure S2 Southern blot analysis of the three GFP-TIAR founders. Genomic DNA was extracted from the tails of wild-type and transgenic mice. The DNAs were digested by BamHI and probed with a TIAR DNA probe revealing endogenous and transgenic TIAR sequences. (1: WT; 2: alpha; 3: beta; 4: gamma strain).
(2.11 MB TIF)
