# Supplementary figures and images for: Impaired Embryonic Development in Mice Overexpressing the RNA-Binding Protein TIAR
Source: PLoS One. 2010 Jun 28;5(6):e11352. doi: 10.1371/journal.pone.0011352 (PMC2893167; doi:10.1371/journal.pone.0011352)

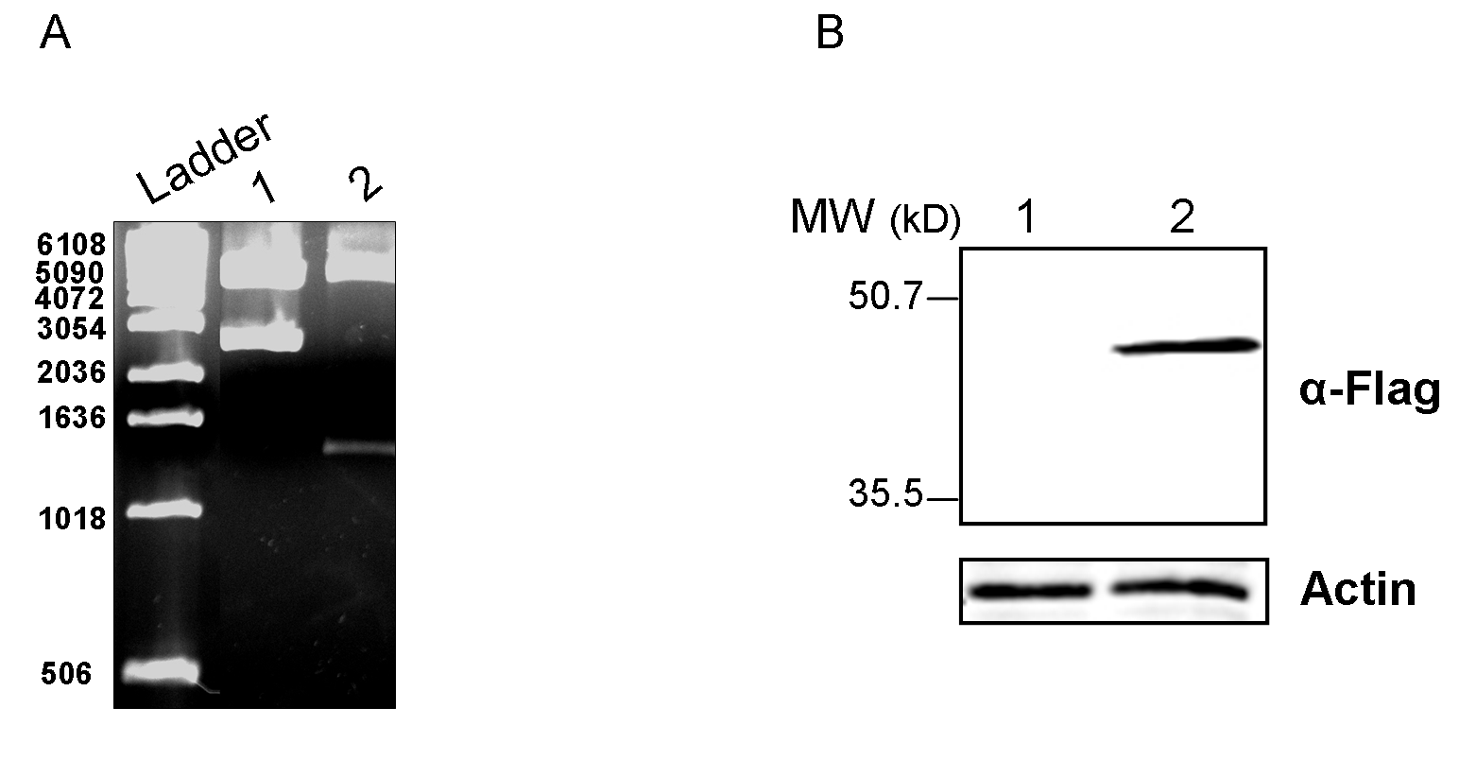

Supplement: Figure S1 — Characterization of TIAR transgene. (A) Transgene recombination in Cre recombinase-expressing bacteria. Plasmid DNA was transformed and amplified in wild-type or Cre recombinase-expressing bacteria (294-Cre) (gift of S. Schurmans, ULB). Plasmid DNA was then isolated and analyzed by agarose gel electrophoresis after cleavage by Hind III restriction enzyme. The low molecular weight fragment generated by HindIII cleavage is 1385 bp shorter in Cre recombinase-expressing bacteria (lane 2) than with plasmid DNA isolated from wild-type bacteria (lane 1). The size (bp) of the fragments composing the ladder is indicated. (B) Plasmid DNAs amplified in wild-type or Cre recombinase-expressing bacteria were transiently transfected into COS cells using Fugene-6 (Roche) according to the manufacturer's instructions. Cells were harvested 48 h after transfection and lysed for western blot analysis of TIAR-Flag expression with anti-Flag antibody (upper panel). Twenty µg of cell extract was loaded on the gel. The membrane was subsequently incubated with anti-actin antibody to control gel loading (lower panel). (3.34 MB TIF) [file pone.0011352.s001.tif]

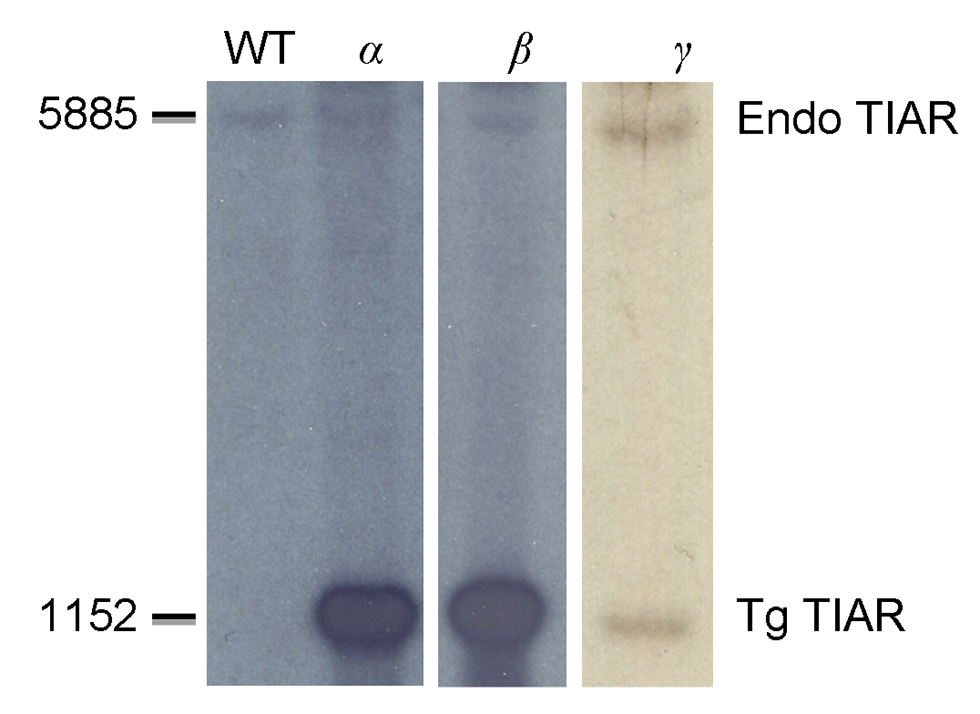

Supplement: Figure S2 — Southern blot analysis of the three GFP-TIAR founders. Genomic DNA was extracted from the tails of wild-type and transgenic mice. The DNAs were digested by BamHI and probed with a TIAR DNA probe revealing endogenous and transgenic TIAR sequences. (1: WT; 2: alpha; 3: beta; 4: gamma strain). (2.11 MB TIF) [file pone.0011352.s002.tif]
